# Multisensory stimulation and rehabilitation for disability improvement: Lessons from a case report

**DOI:** 10.1097/MD.0000000000031404

**Published:** 2022-11-18

**Authors:** Viviana Lo Buono, Michele Torrisi, Simona Leonardi, Alessandra Pidalà, Francesco Corallo

**Affiliations:** a IRCCS Centro Neurolesi Bonino Pulejo Messina, Italy.

**Keywords:** multisensory environment, rehabilitation, spastic quadriplegia

## Abstract

Spastic quadriplegia is most severe form of Infantile Cerebral Palsy. Patients are unable to use their legs, arms and body and show language disorder and profound intellectual disability. The treatment of patients diagnosed with spastic quadriplegia is complex and multidisciplinary. In this case report we described the positive effect of multisensory environment (MSEs) rehabilitation, and the strategies and technologies used to provide child who have to severe spastic quadriplegia and intellectual disability, palsy with playful and fun activities designed according to his abilities.

**Patient concern:**

A 7-years-old boy diagnosed with spastic quadriplegia and severe intellectual disability began rehabilitation by MSEs.

**Diagnoses:**

Spastic quadriplegia is most severe form of Infantile Cerebral Palsy. Patients are unable to use their legs, arms and body and show language disorder and profound intellectual disability.

**Interventions:**

Multisensory room is a large environment containing various elements where child can interact spontaneously and independently.

**Outcomes:**

The comparison scores between *T*0–*T*1 showed a reduction in self-harm and motor stereotypies (hand flapping). Sustained attention was improved and we observed a better therapeutic compliance by means of greater involvement in gaming activities.

**Conclusion:**

The stimuli within the MSEs provided the child opportunities to express himself with facilities more suited to his potential. Future research should project designed randomized controlled trials to examine the efficacy of multisensory on reduction disability.

## 1. Introduction

Infantile Cerebral Palsy (CP) is a non-progressive disorder of the posture and movement, frequently associated with epilepsy, speech, vision sensory deficits, behavior, and intellect disorders.^[1]^ CP that occurs with microcephaly, spasticity, and profound mental retardation is usually due a perinatal asphyxia or congenital infection.^[2]^

Spastic quadriplegia is the most severe form of CP where patient is unable to use their legs, arms and body. Symptoms of spastic quadriplegia can include: lack of muscle coordination when performing voluntary movements; stiff or tight muscles and exaggerated reflexes; weakness in arms or legs; shaking (tremor) or random involuntary movements; speech and language disorder, seizures, cognitive disabilities.^[3]^ The treatment of patients diagnosed with spastic quadriplegia is complex and multidisciplinary. The therapeutic approach, including medications, physiotherapy, speech therapy and occupational therapy, aimed to achieve an adequate functional status of the child.^[4]^ In recent years, controlled multisensory stimulation therapy in children with spastic tetraplegia has shown positive effects on the speed of motor responses^[5]^ and communication.

This case report examined the effect of multisensory environment (MSEs) rehabilitation in a pediatric patient with spastic quadriplegia and intellectual disability.

## 2. Case presentation

We report the case of a 7-year-old boy diagnosed with spastic quadriplegia and severe intellectual disability. He was born at 28 weeks gestational age with severe respiratory failure and he weighed 1.40 kg. In the various stages of development, the child presented with severe psychomotor delay and growth retardation (walking and verbal language not acquired, sphincter control absent), psychomotor restlessness, repetitive behaviors (stereotypies and self-harm). From the age of 3 he was kept on regular treatment with risperidone (1 mg/day).

In September 2020, he began rehabilitation with motor and cognitive training at a clinical rehabilitative center. Clinical assessment was carried out at admission to neurocognitive training (*T*0) and after 2 months (*T*1). At *T*0 the neurological examination showed absence of eye contact, motor stereotypies consisting of hand flapping or twisting, body rocking, signs of self-harm to the hands and face, spastic quadriplegia, muscular hypertonicity s in the four limbs, mainly in the lower limbs, hyperevocable ROT in the lower limbs. Gross Motor Function Classification System LEVEL V. Manual Ability Classification System LEVEL V. Pediatric Functional Independence Measures were used to provide an indication of functional outcomes.

We also used the Vineland Adaptive Behavior Scales, a standardized parent interview to assess adaptive behavior.

The rehabilitative program was administered by a psychologist and a physiotherapist for a period of 1 month 2 times per week for a total of 12 sessions. Each rehabilitative session was composed as follows: motor exercises centered on flexibility exercises and stretching out stiff muscles (50 minutes), sensorial and cognitive stimulations in MSEs room (60 minutes).

**Figure 1.**
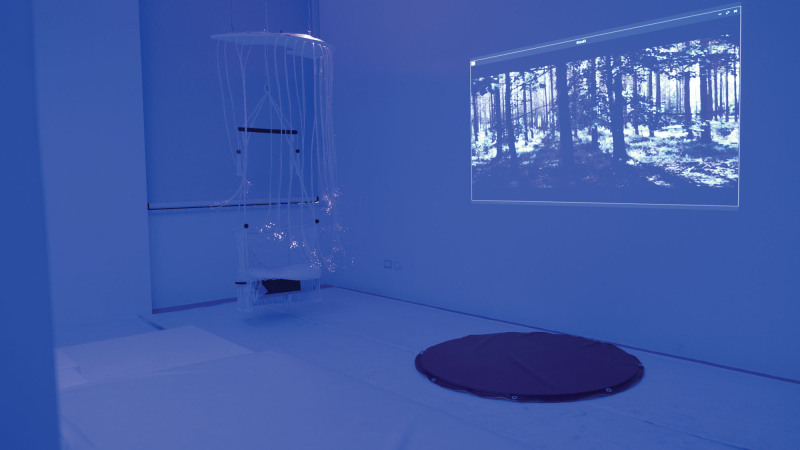
Multisensory room.

Multisensory room is a large space containing various elements where child can interact spontaneously and independently. There is a screen that projects music and relaxing images, a water mattress, a bath with colored balls, an obstacle path, a luminous swing, and several lamps. By touching a button on the wall, it is possible to change the color of the light in the room and its elements (see Fig. 1).

Therefore, child can feel visual, tactile, auditory, and proprioceptive sensory experiences. Although the standard approach of the MSEs is non-directive allowing the patient to freely explore the room in this case, due to the child’s severe motor and cognitive disabilities, the therapist conducted the activities in a direct manner.

Each therapeutic session in MSEs consisted of 2 phases: relaxing and stimulation. The first phase, lasting approximately 20 minutes, was dedicated to physical and mental relaxation through the sound of streams or waterfalls, music, and soft lights. In the next forty minutes, the therapist encouraged interaction of the child with the elements of the room.

The comparison scores between *T*0–*T*1 showed a reduction in self-harm and motor stereotypies (hand flapping). The times of sustained attention was improvement and we observed a better therapeutic compliance by means of greater involvement in gaming activities. Clinical scores are summarized in Table 1 and Table 2.

**Table 1:** Pediatric Functional Independence Measure (WeeFIM) score before (*T*0) and after (*T*1) rehabilitation sensory training.

*Domains 1: self-care*	*T*0	*T*1
*Eating*	1	1
*Grooming*	1	1
*Bathibg*	1	1
*Dressing-upper*	1	1
*Dressing-lower*	1	1
*Toileting*	1	1
*Bladder*	1	1
*Bowel*	1	1
*Domains 2: mobility*		
*Chair transfer*	1	2
*Toilettransfet*	1	1
*Tubtrasfer*	1	1
*Walk*	1	1
*Stairs*	1	2
*Domains 3: cognition*		
*Comprehension*	3	4
*Expression*	3	4
*Social interaction*	3	5
*Problem solving*	1	2
*Memory*	2	2
***Total scores***	**25**	**32**

WeeFIM = pediatric functional independence measures.

**Table 2:** Vineland-II adaptive behavior profile before (*T*0) and after sensory stimulations (*T*1).

DOMAIN	*T*0	*T*1
*Communication*		
*Receptive*	1	1
*Expressive*	1	1
*Written*	1	1
*Daily Living Skills*		
*Personal*	1	1
*Domestic*	1	1
*Community*	1	1
*Social Skills*		
*Interpersonal Relationships*	1	2
*Play and Leisure Time*	1	2
*Coping Skills*	1	1

## 3. Discussion

MSEs or “Snoezelen room” is a form of therapy which uses a combination of environmental factors, such as music and lights, and techniques such as calming touch and movement to provide relaxing sensory stimulation.^[6,7]^ The access to a sensory room provides a variety of benefits that will likely vary for each individual because each person has different sensitivities and ways of reacting to sensory stimulations. Reported positive outcomes of MSEs use include decrease in agitation, better pain management, improved attention time, and a reduction in maladaptive behaviors.^[8]^ Some authors^[9,10]^ have applied a multisensory stimulation for children or adults with a range of severe motor disabilities, including quadriplegia but have not used a “Snoezelen room” as setting.

In child, although the clinical picture remained severe, after rehabilitation training, we observed an improvement of his gross motor skills and non-verbal communication. The stimuli within the MSEs provided the child opportunities to express himself with facilities more suited to his potential. For example, through purposeful behavior (clap the hands) a child has learned to express his preference for a particular music. By touching the objects, he learned to distinguish smooth surfaces from rough ones.

The systematic development of multisensory methods for people with severe disabilities has only recently received much attention although it seems to represent a clinical approach with positive effects on behavior disorders, social interactions and cognitive deficit.^[11]^ The acceptance of this method should be accompanied by a series of research efforts to formally evaluate its effectiveness with respect to cognitive behavioral interventions.^[12]^ On the other hand, however, valid empirical research is limited due to methodological problems such as the difficulty in measuring outcomes. To date is not well known about how children and adolescents who have limited verbal and mobility capacity experience this form of sensory stimulation.^[13]^

Future research should project designed randomized controlled trials to examine whether and how multisensory stimulation can aid reduction of disability.

## Author contributions

**Conceptualization:** Viviana Lo Buono.

**Data curation:** Francesco Corallo.

**Investigation:** Simona Leonardi, Alessandra Pidalà.

**Methodology:** Francesco Corallo.

**Project administration:** Viviana Lo Buono.

**Supervision:** Francesco Corallo, Michele Torrisi, Alessandra Pidalà.

**Validation:** Michele Torrisi, Alessandra Pidalà.

**Visualization:** Simona Leonardi, Viviana Lo Buono

**Writing – original draft:** Viviana Lo Buono.

**Writing – review & editing:** Michele Torrisi.
